# A psychological perspective on peer tutoring in EFL collaborative writing in Chinese higher education contexts

**DOI:** 10.1371/journal.pone.0362263

**Published:** 2026-11-06

**Authors:** Zefu Huang, Nalini Arumugam

**Affiliations:** 1 School of Foreign Studies, Anhui Polytechnic University, Wuhu, China; 2 Faculty of Education, Language, Psychology and Music, SEGi University, Petaling Jaya, Malaysia; Bahir Dar University, ETHIOPIA

## Abstract

In Chinese higher learning institutions, students learning English as a foreign language (EFL) often demonstrate low writing self-efficacy (WSE) and poor writing competence. The present study examined the associations between the integration of peer tutoring with collaborative writing and students’ WSE and writing outcomes from a psychological perspective. A quasi-experiment was implemented for 11 weeks for 100 Chinese university students studying EFL. Changes in WSE were examined, and writing competence was assessed using tests. The results showed a notable rise in students’ WSE after the instructional intervention. The multiple linear regression analysis indicated that gains in WSE were positively associated with the writing outcome after controlling for pretest writing competence and group membership, explaining 20.2% of the total variance. These results suggest that peer-supported collaborative writing was associated with higher EFL writing competence alongside increased WSE. These findings align with theoretical justifications based on peer scaffolding and the Zone of Proximal Development, although these processes were not directly measured in the present study. These findings imply that incorporating peer tutoring into collaborative writing might be associated with the development of WSE and improved writing competence among EFL students at Chinese universities. Increased WSE appeared to be a significant, albeit partial, predictor of writing competence, highlighting the potential relevance of WSE development in peer-supported collaborative writing contexts.

## Introduction

Learning how to write well, especially for students of English as a foreign language (EFL), is not only about improving language skills; it is a complicated dynamic psychological and cognitive interaction process [1]. In the case of China’s EFL classrooms, two interrelated problems that tend to occur and affect students’ language acquisition are: low levels of writing self-efficacy (WSE) and underdeveloped skills and strategies for producing effective texts [2,3]. According to Bandura [4], self-efficacy means a fundamental confidence that is associated with an individual’s task involvement and overall performance. Regarding EFL students, WSE played an important role in supporting their overall writing competence [5,6].

Peer tutoring and collaborative writing are two widely investigated approaches employed in EFL writing courses to help students become more proficient writers. Peer-supported learning approaches, including peer instruction and peer tutoring, share the common emphasis on active learner engagement and knowledge exchange. Although Mazur’s [7] peer instruction was originally developed in physics education, related peer-supported principles have informed student-centered instructional practices in EFL contexts. Peer tutoring draws on principles of peer instruction, which can shift traditional teacher-centered classrooms into dynamic student-centered ones that emphasize involvement, negotiation, and inquiry. This approach actively inspires students to exchange their viewpoints and thoughts with peers and, more importantly, to offer scaffolding to relatively less proficient peers [8,9]. In collaborative writing, the interactive process of discussing ideas, negotiating meaning, and jointly constructing text also enhances cognitive processes in essay composition [10].

While educational research has documented the general benefits of peer tutoring and collaborative learning for language acquisition, an important psychological perspective remains insufficiently explored. Most studies in the EFL context have primarily evaluated writing competence through overall proficiency scores [11–13]. Few have examined how changes in writing competence relate to gains in WSE. Therefore, the present study aims to ascertain whether EFL students’ changes in WSE were positively associated with writing competence following peer tutoring integrated with collaborative writing in Chinese higher institutions.

## Literature review

### Peer tutoring

In 1997, Eric Mazur of Harvard University developed and improved the peer instruction approach. Prior to this, Mazur, who had been teaching physics since 1984, adopted conventional instructional strategies such as assigning textbook homework, delivering lectures, conducting experiments, and reviewing material from textbooks [14]. Nevertheless, in the 1990s, he began to recognize the shortcomings in the conventional teacher-centered approaches. Students often attended the classes with already-known ideas about physics derived from their daily experiences. These concepts were often incorrect, making it difficult for them to grasp the scientific notions their teachers were trying to convey, hindering improvements in students’ scientific understanding and the correction of their stereotypes about science under traditional teaching methods. To tackle that problem, Mazur [7] proposed a new teaching approach called peer instruction.

Peer instruction was initially introduced into China by Zhang and Mazur [15] through their paper “Peer-instruction: A new method of physics teaching in Harvard University”, which was published in a journal called *China University Teaching*. In that paper, the peer instruction approach was described in detail, with an extensive discussion of how it could be used in pedagogical practice. Peer tutoring and peer instruction are different but interrelated forms of peer-assisted learning. Peer instruction emphasizes peer discussion and interaction to solve conceptual problems in particular disciplines, originally physics. However, peer tutoring involves structured peer support, guidance and feedback to promote overall improvement in learning. Recently, peer tutoring has been adopted by Chinese teachers from various educational levels [14].

The peer tutoring approach has a solid theoretical basis in Vygotsky’s concept of the Zone of Proximal Development (ZPD). It was claimed that children experience two distinct phases of development: current and prospective [16]. The current developmental level reflects the psychological processes resulting from a particular existing capability of an individual. In contrast, the prospective development level refers to the potential level that students have not yet reached. Vygotsky [16] introduced the concept of the ZPD to describe the gap between these two levels. According to him, through adult or peer scaffolding, children’s ZPD can be expanded, and they have a higher level of potential to achieve. Based on Vygotsky’s ZPD principle, students can develop and enhance their writing competence through peer scaffolding [17,18].

Despite its documented pedagogical benefits and wide applications, research on peer tutoring in Chinese EFL writing remains predominantly focused on its effectiveness on writing skills and outcomes [19]. A considerable gap exists in the empirical examination of how peer tutoring and collaborative writing might be associated with writing competence from a psychological perspective.

### Collaborative writing

Collaborative writing is the process in which a group of students cooperate and contribute to create a work of writing, and the final product is expected to be superior to that of an individual student [20]. The following is a summary of the advantages of collaborative writing, as outlined by Storch [21]. It first promotes learning and cognitive growth in students. Through collaborative writing, students develop greater awareness of their listeners’ understanding and become reflective thinkers. Additionally, collaborative writing encourages students’ use of language in output, including lexical and grammatical choices.

Numerous benefits of collaborative writing in EFL writing lessons have been demonstrated by research. It can assist students in considering how they utilize language when writing. Furthermore, collaborative writing improves all four fundamental English language writing abilities [22]. In addition, studies have demonstrated that collaborative writing lowers learning anxiety and raises students’ WSE [23]. Usher et al. [24] found that students’ improved writing competence also inspired them to write more and learn more.

The advantages of collaborative writing are clear from the results of earlier studies. Language students can improve their accuracy and competence in language, particularly in writing, by participating in collaborative writing exercises that allow them to see and debate language forms in context [25]. Through collaborative scaffolding provided by collaborative writing, students can enhance their learning and second-language acquisition. As a consequence, collaborative writing assists students in developing their skills, in sharing their own ideas, and in accepting others’ viewpoints [26].

Nevertheless, existing studies have primarily regarded writing competence as an integrated concept, measured by scores derived from specific rubrics. Furthermore, there is a lack of investigation into how the interactive processes within collaborative writing—such as idea negotiation and peer scaffolding—specifically influence and engage with students’ psychological attributes.

### WSE among EFL students

Bandura [4] defined self-efficacy as an individual’s belief in his or her capacity to fulfil a specific assignment. Many studies have demonstrated that this lack of WSE primarily results from students’ uncertainty about their abilities and their high expectations stemming from the given writing standards [27,28]. In Chinese EFL classrooms, students encounter considerable challenges in writing [29]. When students write, they often feel nervous, anxious, or even depressed. They worry that they won’t meet the high standards. This negative emotional state stops them from improving their writing skills [5,30].

The deficiency in the foundation of the English language is undoubtedly a significant factor related to EFL students’ low WSE [6,31]. Nevertheless, the conventional teacher-centered teaching approaches also prevent EFL students’ WSE. These approaches often excessively emphasize grammar and vocabulary accuracy, causing students to overlook the creativity of overall structure and content [32]. Studies have demonstrated that within such teaching frameworks, students struggle to cultivate stable, high-level WSE and instead experience excessive anxiety about making mistakes in writing, which prevents them from exploring diverse and creative writing styles and techniques [33,34]. Recent research further suggests that WSE can be influenced by instructional environments. For example, Talebzadeh et al. [35] found that different instructional modes affected EFL learners’ writing motivation and WSE, indicating that WSE is responsive to pedagogical contexts.

Although earlier research has addressed the reasons behind low WSE, little is known about interventions that examine how pedagogical innovations, such as peer tutoring combined with collaborative writing, can actively increase students’ WSE and, ultimately, writing competence from a psychological standpoint. The present study attempts to fill the research gap by examining the association between collaborative writing integrated with peer tutoring and EFL students’ writing competence from a psychological perspective.

## Methods

### Research design

As shown in Table 1, the present study followed a quasi-experimental design. Quasi-experimental designs offer researchers flexibility to conduct experimental research in real-world conditions when it is not feasible to control all potential variables that can influence the study [36,37].

**Table 1:** Quasi-experimental research design.

Group	Pre-survey	Pretest	Intervention	Post-survey	Posttest
EG	O_1_	O_2_	X	O_3_	O_4_
CG	O_1_	O_2_	—	O_3_	O_4_

**Note.** — = no intervention. X = peer tutoring in collaborative writing. O_1_ = WSE in pre-survey; O_2_ = writing competence in pretest; O_3_ = WSE in post-survey; O_4_ = writing competence in posttest.

The present study selected two intact EFL learner groups, the experimental and control groups (hereafter the EG and the CG), from the same Chinese university. Before the intervention, pretests of writing competence and pre-surveys of WSE were administered to both groups, serving as the baseline measurements for the study. During the 11-week intervention, participants in the EG received peer tutoring integrated with collaborative writing in their English writing courses. In the CG, students had their writing instruction as normal, with no peer tutoring or collaborative writing. The overall research procedure is presented in Fig 1.

**Fig 1:**
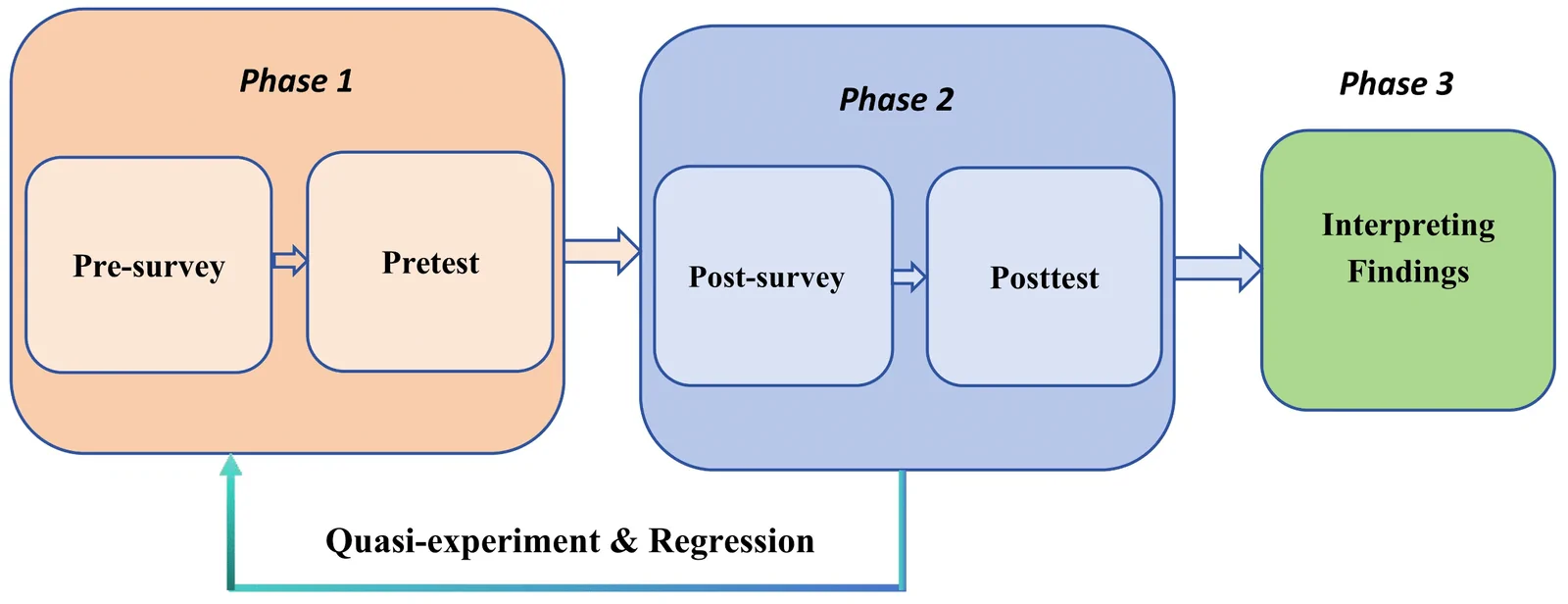
Flowchart of the research design.

The present study expected that, after the intervention, the participants would have an improved WSE, which might be associated with enhanced writing competence. Therefore, all participants in both groups completed post-surveys and posttests to assess their WSE and writing competence after the intervention. By observing and comparing the data collected from both groups before and after the intervention, the present study examined the relationship between shifts in WSE and writing competence following peer tutoring integrated with collaborative writing.

### Participants

On February 14, 2025, convenience sampling was utilized to select two existing intact classes from a public university in eastern China, taking both the instructor’s availability and the class timetable into account. The participants included 100 sophomore non-English major students (Average Age = 19.6 years, *SD* = .7; 52 females, 48 males) who were enrolled in an optional college English class. They represented diverse disciplinary backgrounds, including textile engineering, applied chemistry, computer science, new energy, and robotics. The two intact classes were designated as the EG (*n* = 50) and the CG (*n* = 50), following the university’s overall teaching schedule. It was not appropriate to adopt individual random sampling because the present study was conducted in a real classroom teaching environment. The allocation was determined by the existing class schedule rather than by random assignment.

Baseline equivalence between the two groups was examined for initial writing competence and WSE, with no statistically significant differences. Therefore, the findings suggest baseline comparability between groups.

No formal recruitment was needed for the present study, as all selected participants came from two intact classes. Before they were involved, all the participants signed informed consent documents outlining their freedom to withdraw at any time, the fact that participation is entirely voluntary, and the confidentiality of their data. In addition, all procedures were carried out in compliance with ethical rules, and the present study was approved by the Ethics Committee of Institute of Neuroscience and Cognitive Psychology of AHPU (Ethics review number: AHPU-SLS-2025–003).

### Research instruments

#### Pretest and posttest.

The participants took a preliminary assessment (pretest) excerpted from the writing section of the CET-4 examination before the intervention. The preliminary assessment for students was to write a short 150-word composition describing a book they had read recently. The writing competence was initially scored on a 15-point scale based on the CET-4 writing scoring criteria and evaluated across four dimensions: content, organization, language, and conventions. The raw scores were then linearly converted to a 100-point scale for analysis. Prior to the formal assessment, the evaluation criteria were discussed with three English instructors (raters) to ensure a shared understanding of the rubric and grading procedures. During the grading process, the three trained raters scored all writing samples independently and blindly. They were unaware of the participants’ group assignments. Neither did they identify whether these compositions were collected before or after the intervention. The intraclass correlation coefficient (ICC) was adopted to examine the inter-rater reliability. It was analyzed through a two-way random-effects model characterized by an absolute agreement definition. The ICC, for all 100 pretest writing samples, indicated a value of .614 for single measures (95% CI [.513, .721]) and .827 for average measures (95% CI [.760, .882]). They both demonstrated acceptable to good inter-rater reliability. After 11 weeks of instructional intervention, participants took a second assessment (posttest) with the same writing prompt, intentionally used to ensure comparability, although potential task familiarity effects were acknowledged. The ICC for all 100 posttest writing samples was .720 for single measures (95% CI [.637, .792]) and .885 for average measures (95% CI [.840, .919]). The final writing score was calculated as the mean of the three raters’ scores. As the writing assessment was based on the CET-4 writing scoring criteria, the use of an established national assessment framework provided a standardized basis for evaluating students’ writing competence.

#### Pre-survey and post-survey.

In the present study, the questionnaire was administered before and after the intervention. It consisted of two sections: participants’ background information and a WSE questionnaire. Although established WSE scales were available, the present study adopted a writing-performance rubric [38] as the conceptual basis because its dimensions corresponded to key aspects of English writing performance that could be expressed as students’ perceived writing capabilities. The original writing dimensions were adapted into first-person statements to measure students’ WSE. The adaptation was intended to align the perceived capability items with the specific writing dimensions assessed in the present study. The adapted items were reviewed by three English language education experts for clarity and relevance, and their feedback was used to refine the items before pilot testing. The initial questionnaire consisted of 25 items. Following exploratory factor analysis, 18 items were retained in four dimensions: WSE in content, WSE in organization, WSE in language use, and WSE in mechanics, with all items rated on a 7-point Likert scale.

Sample items included: “I can make reasonable choices and support my viewpoint” (content); “I can use transitional words and sentences to connect paragraphs and sentences, making the article more fluent” (organization); “I can identify and correct complex grammar errors, such as connecting clauses and emphasizing the use of sentence structures” (language use); “I can follow the norms of English writing, such as spelling, punctuation, and capitalization” (mechanics). The complete 18 items in the WSE questionnaire have been provided in the “Supporting information” files, entitled “S2 File”.

A pilot study was carried out to examine the instrument’s reliability and factor structure before its administration in the formal experiment. A sample of 133 students was involved. They had characteristics comparable to the target participants in the formal experiment. The overall Cronbach’s alpha coefficient of the questionnaire was .965, indicating great internal consistency. As shown in Table 2, exploratory factor analysis using principal component analysis with Varimax rotation supported the four-dimensional structure, with KMO = .937 and Bartlett’s test of sphericity, χ² (153) = 2235.553, p < .001. The four components accounted for 78.958% of the total variance. The overall Cronbach’s alpha coefficient of the final 18-item questionnaire was .965, with coefficients of .935, .886, .927, and .903 for content, organization, language use, and mechanics, respectively.

**Table 2:** Exploratory factor analysis and reliability results for the WSE scale.

Dimension	Retained Items	No. of Items	Factor Loading	Cronbach’s *α*
Content	1, 2, 3, 4, 5	5	.696–.829	.935
Organization	7, 8, 9, 18	4	.620–.750	.886
Language Use	12, 13, 15, 16, 17, 19	6	.603–.736	.927
Mechanics	23, 24, 25	3	.708–.798	.903
Overall WSE	18 retained items	18	.603–.829	.965

Note. The initial WSE scale contained 25 items. Exploratory factor analysis resulted in the retention of 18 items, with 7 items removed. KMO = .937; Bartlett’s test of sphericity, χ² (153) = 2235.553, *p* < .001. A four-component solution was examined using principal component analysis with Varimax rotation, accounting for 78.958% of the total variance.

### Intervention procedures

The intervention for the EG took place in a college English writing classroom over 11 weeks. The EG and the CG followed the same syllabus, engaged in identical writing tasks, and received equal teaching time (one teaching session per week). The teaching content covered four major writing genres: narration, description, exposition, and argumentation.

In addition, to minimize potential selection bias, both groups received instruction from the same instructor with identical instructional materials. They were exposed to equivalent learning conditions. The same writing rubric and scoring procedures were used to maintain consistency across groups. Before the intervention, students in the EG received an introductory session of training on the procedures and expectations of peer tutoring. To support consistency across the 11-week intervention, the instructor followed standardized teaching plans that specified the instructional content, activities, and procedures for each week. Detailed 11-week teaching plans for both groups are provided as the S3 and S4 Files in the “Supporting information” files.

The primary difference between the two groups was the intervention of peer tutoring in collaborative writing in the EG. Specifically, in this group, instruction followed a structured process writing cycle consisting of planning, drafting, and revision. In each stage, students worked in fixed peer groups of four, with one group leader in each group. The groups were randomly formed and remained unchanged throughout the 11-week intervention to reduce free-riding. Students provided structured feedback on content, organization, language use, and mechanics with the help of the teacher’s guiding prompts. Finally, each group submitted one joint piece of writing. Peer tutoring referred to structured assistance among group members through explaining, questioning, clarifying, and correcting peers’ ideas, while peer feedback referred to comments and suggestions provided on peers’ drafts; collaborative writing involved joint planning, drafting, editing, and revising.

The CG, on the other hand, was given traditional writing instruction. Although students were exposed to the same writing genres and sample essays, their learning activities primarily involved individual writing, teacher explanation, and instructor feedback. No structured peer tutoring or guided peer feedback procedures were implemented in the CG.

These procedures were designed to reduce the potential sources of variation between the two groups, with the primary instructional difference being the presence or absence of peer tutoring integrated with collaborative writing.

### Data collection and analysis

The data of the present study were accessed from the beginning to the end of the quasi-experiment (February 17 to May 28, 2025) for research purposes only. The participants’ personal information (names and student IDs) was temporarily accessible during the data collection period due to assigning small writing groups and matching scores between pretest and posttest, and between pre-survey and post-survey. However, after data collection, all data were anonymized to protect participants’ confidentiality by removing their names and student IDs.

During the data analysis, SPSS (Version 27) was used. First, data were screened to ensure there were no missing values or outliers. Then, a series of statistical analyses was conducted, including a normality test, an independent-samples *t*-test, ANCOVA, and multiple regression analysis. The results of the data analysis are displayed in the next chapter.

## Results

### Normality test

To determine if the data were normally distributed, the Shapiro-Wilk test was employed [39]. As shown in Table 3, in the pre-survey, the *p*-value of the normality test was .149 for the EG and .058 for the CG. In the post-survey, the *p*-values were .422 and .073 for the EG and the CG, respectively. All *p*-values were greater than the .05 threshold, indicating that the WSE scores were distributed with no significant departures from normality in both groups at both pre-survey and post-survey.

**Table 3:** Normality test of WSE scores.

Variable	Group	Kolmogorov-Smirnov			Shapiro-Wilk		
		Statistic	df	Sig.	Statistic	df	Sig.
WSE_pre (Pre-survey)	EG	.107	50	.200	.965	50	.149
	CG	.121	50	.063	.956	50	.058
WSE_post (Post-survey)	EG	.086	50	.200	.977	50	.422
	CG	.123	50	.055	.958	50	.073

The normality test results for the writing scores before and after the intervention are given in Table 4. The *p*-values of the pretest were .214 and .103 for the EG and the CG, respectively. In the posttest, these were .176 and .104. All the probability values were above the common .05 cutoff, and therefore, the writing scores maintained normal distribution properties. Consequently, the basic requirements for performing parametric statistical analyses were met for all measured variables.

**Table 4:** Normality test of writing scores.

Variable	Group	Kolmogorov-Smirnov			Shapiro-Wilk		
		Statistic	df	Sig.	Statistic	df	Sig.
pretest	EG	.124	50	.054	.969	50	.214
	CG	.122	50	.060	.962	50	.103
posttest	EG	.123	50	.056	.967	50	.176
	CG	.106	50	.200	.962	50	.104

### WSE outcomes

The present study conducted independent-samples *t*-tests to compare the WSE scores of the EG and the CG in both the pre-survey and post-survey to investigate whether peer tutoring integrated with collaborative writing was associated with differences in EFL students’ WSE.

#### Initial WSE assessment.

The two groups’ baseline WSE levels were compared by utilizing the independent-samples *t*-test. Table 5 showed that the mean WSE score was 4.269 (*SD* = .203) for the EG and 4.253 (*SD* = .190) for the CG. In addition, there was no difference at the start between the EG and the CG, *t*(98) = .397, *p* = .692, mean difference = .016, 95% CI [−.062, .094], Cohen’s *d* = .079. This result indicates that the EG and the CG did not differ significantly at baseline on WSE.

**Table 5:** Independent-samples *t*‑test for pre-survey WSE.

Variable	Group	*M* ± *SD*	*t*(df)	*p*	Mean Difference	95% CI	Cohen’s *d*
WSE_pre	EG	4.269 ± .203	.397 (98)	.692	.016	−.062 to .094	.079
	CG	4.253 ± .190					

#### Post-survey WSE evaluation.

The same parallel examination was conducted for students’ WSE levels, measured in the post-survey. Table 6 shows that the mean WSE score was significantly higher in the EG (*M* = 4.542, *SD* = .310) than in the CG (*M* = 4.303, *SD* = .231). There was a statistically significant difference between the EG and the CG after the 11-week intervention, *t*(98) = 4.369, *p* < .001, mean difference = .239, 95% CI [.130, .347], Cohen’s *d* = .874. The large effect size indicates that the intervention, peer tutoring integrated with collaborative writing, may be associated with substantial differences in students’ WSE compared with the conventional instructional approach.

**Table 6:** Independent-samples *t*‑test for post-survey WSE.

Variable	Group	*M* ± *SD*	*t*(df)	*p*	Mean Difference	95% CI	Cohen’s *d*
WSE_post	EG	4.542 ± .310	4.369 (98)	< .001	.239	.130 to .347	.874
	CG	4.303 ± .231					

Overall, the results suggest a similar baseline of WSE between the two groups before the intervention started. The post-survey assessments suggest that the EG who received the intervention, peer tutoring integrated with collaborative writing, obtained much higher WSE scores than the CG.

#### ANCOVA of post-survey WSE.

To directly examine the intervention effect while accounting for baseline variation, an ANCOVA was conducted with post-survey WSE as the dependent variable, group as the between-subjects factor, and pre-survey WSE as the covariate. Table 7 shows that the homogeneity of regression slopes was supported, as the Group × WSE_pre interaction was not significant, *F* (1, 96) = 1.023, *p* = .314. Homogeneity of error variances was also supported by Levene’s test, *F* (1, 98) = 2.867, *p* = .094.

**Table 7:** ANCOVA results for post-survey WSE by group.

Source	*SS*	*df*	*MS*	*F*	*p*	Partial *η*²	Adjusted *M* (*SE*)	95% CI
WSE_post	.063	1	.063	.839	.362	.009	—	—
Group	1.447	1	1.447	19.345	<.001	.166	EG: 4.543 (.039)	[4.466, 4.620]
							CG: 4.302 (.039)	[4.225, 4.379]
Error	7.254	97	.075	—	—	—	—	—
Corrected Total	8.742	99	—	—	—	—	—	—

**Note.** Post-survey WSE was the dependent variable, with pre-survey WSE entered as the covariate. The homogeneity of regression slopes assumption was satisfied, *F* (1, 96) = 1.023, *p* = .314; homogeneity of error variances was also satisfied, Levene’s *F* (1, 98) = 2.867, *p* = .094.

After controlling for pre-survey WSE, the group effect was significant, *F* (1, 97) = 19.345, *p* < .001, partial *η*² = .166. The adjusted mean was higher for the EG (*M* = 4.543, *SE* = 0.039, 95% CI [4.466, 4.620]) than for the CG (*M* = 4.302, *SE* = 0.039, 95% CI [4.225, 4.379]). The pre-survey WSE covariate was not significant, *F* (1, 97) = 0.839, *p* = .362. Thus, after accounting for baseline WSE, the EG showed significantly higher post-survey WSE than the CG.

### Writing competence outcomes

To examine differences in writing competence between the EG and the CG after the intervention, the present study also employed independent-samples *t*-tests to compare the differences in writing scores between the EG and the CG in both the pretest and the posttest.

#### Initial writing competence assessment.

The present study conducted a comparative analysis of pretest writing scores, following an independent-samples *t*-test. Table 8 shows that the mean pretest writing score was 71.26 (*SD* = 3.50) for the EG and 71.18 (*SD* = 3.17) for the CG. There was no statistically significant difference in writing competence between the EG and the CG in the pretests, *t*(98) = .120, *p* = .905, mean difference = .080, 95% CI [−1.245, 1.405], Cohen’s *d* = .024. These results indicate that both groups had similar writing competence levels on the pretests, thereby supporting subsequent comparative analyses. The minimal effect size indicates that the EG and the CG were comparable in writing competence before the intervention. This provides a robust baseline for subsequent analyses.

**Table 8:** Independent-samples t-test for pretest writing competence.

Variable	Group	*M* ± *SD*	*t*(df)	*p*	Mean Difference	95% CI	Cohen’s *d*
Pretest	EG	71.26 ± 3.50	.120 (98)	.905	.080	−1.245 to 1.405	.024
	CG	71.18 ± 3.17					

#### Writing competence after intervention.

The same statistical evaluation was conducted on the posttest results. Table 9 shows that the mean posttest writing score was 75.160 (*SD* = 4.372) for the EG and 71.920 (*SD* = 3.355) for the CG. The *t*-test results indicate a statistically significant difference between the two groups, *t*(98) = 4.157, *p* < .001, mean difference = 3.240, 95% CI [1.693, 4.787], Cohen’s *d* = .831. The large effect size indicates that, following the intervention, the EG achieved significantly higher writing competence than the CG. This suggests that peer tutoring integrated into collaborative writing had a substantial association with EFL writing competence.

**Table 9:** Independent-samples t-test for posttest writing competence.

Variable	Group	*M* ± *SD*	*t*(df)	*p*	Mean Difference	95% CI	Cohen’s *d*
Posttest	EG	75.160 ± 4.372	4.157 (98)	< .001	3.240	1.693 to 4.787	.831
	CG	71.920 ± 3.355					

Overall, both groups had similar writing competence in the pretest, but after 11 weeks of intervention of peer tutoring integrated with collaborative writing activities, the EG had higher writing scores in the posttest.

#### ANCOVA of posttest writing performance.

To directly examine the intervention effect while accounting for baseline variation, an ANCOVA was conducted with posttest writing performance as the dependent variable, group as the between-subjects factor, and pretest writing performance as the covariate. Table 10 shows that homogeneity of regression slopes was supported, as the Group × Pretest writing performance interaction was not significant, *F* (1, 96) = 1.219, *p* = .272. Homogeneity of error variances was also supported by Levene’s test, *F* (1, 98) = 2.883, *p* = .093.

**Table 10:** ANCOVA results for posttest writing performance by group.

Source	*SS*	*df*	*MS*	*F*	*p*	Partial *η*²	Adjusted *M* (*SE*)	95% CI
Pretest writing performance	13.698	1	13.698	.901	.345	.009	—	—
Group	263.854	1	263.854	17.355	<.001	.152	EG: 75.164 (.551)	[74.070, 76.259]
							CG: 71.916 (.551)	[70.821, 73.010]
Error	1474.702	97	15.203	—	—	—	—	—
Corrected Total	1750.840	99	—	—	—	—	—	—

**Note.** Posttest writing performance was the dependent variable, with pretest writing performance entered as the covariate. The homogeneity of regression slopes assumption was satisfied, *F* (1, 96) = 1.219, *p* = .272; homogeneity of error variances was also satisfied, Levene’s *F* (1, 98) = 2.883, *p* = .093.

After controlling for pretest writing performance, the group effect was significant, *F* (1, 97) = 17.355, *p* < .001, partial *η*² = .152. The adjusted mean was higher for the EG (*M* = 75.164, *SE* = 0.551, 95% CI [74.070, 76.259]) than for the CG (*M* = 71.916, *SE* = 0.551, 95% CI [70.821, 73.010]). The pretest writing performance covariate was not significant, *F* (1, 97) = 0.901, *p* = .345. Thus, after accounting for baseline writing performance, the EG showed significantly higher posttest writing performance than the CG.

### Association of WSE change with posttest writing competence

The present study also conducted a multiple linear regression analysis to determine whether changes in WSE predicted posttest writing competence, controlling for pretest and group membership. In this regression analysis, the dependent variable was the posttest writing score, and the independent variables were the pretest writing score, group membership, and changes in WSE (WSE_Change). The WSE_Change variable was calculated as the post-survey WSE minus pre-survey WSE.

As demonstrated in Table 11, the multiple regression model was statistically significant, *F*(3, 96) = 8.103, *p* < .001, and explained 20.2% of the variance in posttest writing competence (*R*² = .202, adjusted *R*² = .177). The regression analysis indicated that changes in WSE were a significant positive predictor of posttest writing competence (*β* = .222, *p* = .023) after controlling for pretest writing competence and group membership. Group membership also significantly predicted posttest writing competence (*β* = −.321, *p* = .001). However, pretest writing competence was not a significant predictor (*β*= − .103, *p* = .263), suggesting that baseline writing performance did not make a statistically significant unique contribution to explaining posttest writing competence after accounting for group membership and WSE change. The negative coefficient reflected the coding scheme, where higher values represented the CG.

**Table 11:** Multiple regression analysis predicting posttest writing competence.

Predictor	*B*	*SE*	*β*	*t*	*p*	95% CI for *B*
Constant	86.427	8.320	—	10.388	< .001	[69.912, 102.941]
Pretest	−.130	.116	−.103	−1.126	.263	[−.360, .099]
Group	−2.683	.801	−.321	−3.349	.001	[−4.274, −1.093]
WSE_Change	2.541	1.100	.222	2.309	.023	[.357, 4.725]

Model statistics: *F* (3, 96) = 8.103, *p* < .001, *R*² = .202, adjusted *R*² = .177, *SEE* = 3.815.

**Note.** Groups were coded as 1 = experimental group (EG) and 2 = control group (CG). WSE_Change was calculated as post-survey WSE minus pre-survey WSE, with positive values indicating increases in WSE. Regression diagnostics indicated that linearity and homoscedasticity were reasonably satisfied. The standardized residuals were approximately normally distributed, and no influential cases were identified (maximum Cook’s distance = .103). Collinearity diagnostics indicated no problematic multicollinearity (VIFs = 1.005–1.108).

These findings suggest that greater improvements in WSE were associated with higher posttest writing competence. However, the regression model explained approximately 20.2% of the variance in posttest writing performance (adjusted *R*² = .177), indicating that WSE change made a significant but limited contribution to writing outcomes. A substantial proportion of variance remained unexplained, suggesting that other factors, such as language proficiency, peer interaction quality, task engagement, and individual differences, may also influence writing development. Therefore, WSE development should be considered as one contributing factor rather than the sole determinant of writing achievement in collaborative learning contexts.

## Discussion

The present study examined the psychological factors associated with peer tutoring and collaborative writing activities, and how they are related to Chinese EFL students’ WSE and writing competence.

### Improvement of WSE and writing competence

The independent-samples *t*-test showed that although no significant differences existed in WSE and writing competence between the two groups before the intervention, the EG demonstrated a significantly higher level of WSE and writing competence than the CG after the intervention, which was confirmed by ANCOVA. These results align with previous intervention studies suggesting that pedagogical approaches can enhance students’ self-efficacy and related psychological outcomes [40]. Additionally, similar findings are reported in studies showing that collaborative writing activities reduce academic stress and increase students’ belief in their abilities [23,24,41], and that peer-assisted learning also contributes to higher compositional quality [20,21]. The large effect sizes (Cohen’s *d* = .874 for WSE and .831 for writing competence) indicate that the between-group differences were substantial [42]. These effect sizes are broadly comparable to those reported in previous L2 writing intervention research. A recent meta-analysis of peer-tutoring studies found a large overall effect (Hedges’ *g* = 0.84), while a meta-analysis of collaborative-writing studies reported a large effect for rubric scores (Hedges’ *g* = 0.94) [43,44]. From the perspective of Vygotsky’s [16] ZPD, peer tutoring may provide students with scaffolding and may expand their ZPD, enabling them to achieve the writing goal beyond their own capacities. Such scaffolded learning may reinforce students’ WSE levels and may improve their writing competence.

### WSE’s positive association with writing competence

The statistical analysis indicated that increases in WSE were found to partially predict students’ posttest writing competence. These results provide psychological evidence that peer-supported collaborative writing may be associated with improved writing skills. The integrated approach may enhance students’ WSE, which is associated with their writing competence. This is in line with the broad review by Graham [28] and supports the conclusions of Rahimi and Fathi [45] on collaborative writing, where WSE is positively related to students’ actual writing competence. The multiple regression model explained 20.2% of the variance in posttest writing competence; therefore, although WSE plays a significant but limited predictive role, other factors, such as language proficiency and the effectiveness of peer evaluation, also influence the development of writing skill. Together with the large effect sizes, these findings indicate that the intervention had potential educational value in supporting students’ WSE and writing competence.

### A psychological perspective on peer tutoring in collaborative writing

The findings are consistent with theoretical explanations based on Bandura’s Social Cognitive Theory and Vygotsky’s ZPD theoretical concept. It is important to emphasize that these proposed psychological processes were not directly measured in the present study. The theoretical interpretation offered here should therefore be viewed as a plausible account of the observed findings that requires empirical verification in future research. The observed improvements in WSE and writing competence may reflect psychological processes such as peer scaffolding, observational learning, and collaborative meaning negotiation, which should be examined directly in future research. First, students may share a cognitive burden through peer scaffolding, indicating that writing-related stress is reduced and that difficult assignments are considered achievable goals [46]. Then, observational learning may occur when less skilled writers observe their counterparts who have completed the writing activities, which serves as a powerful source of self-efficacy in Bandura’s [4] social learning theory [47]. Further, the way of developing understanding through meaning negotiation and the collective text is developed, which helps students better understand second-language writing norms, one of the key points in Vygotsky’s [16] ZPD [48]. These elements may create a cycle as follows: when their WSE grows, they have more dedication and perseverance, which in turn may result in their writing competence improving, further strengthening the learner’s belief in their capabilities.

### Limitations and future research

Some limitations of the present study should also be taken into consideration. First, the present investigation relied solely on quantitative data analysis, which may miss subtle aspects of the dynamics of peer learning. The lack of qualitative data further limited the explanation of the psychological mechanisms underlying peer interaction within the ZPD framework, including peer scaffolding, observational learning, collaborative meaning negotiation, and the development of WSE. In future studies, observational techniques or a retrospective interview method could be employed to capture these interaction processes in greater depth. Future research should employ mixed-methods and longitudinal research designs to examine how peer scaffolding, WSE, and writing competence develop over time and across instructional contexts.

Second, the present study employed a convenience sampling technique, and intact classes rather than randomly assigned individuals participated in the quasi-experiment. Although the two groups demonstrated comparable baseline writing competence, intact classes may not eliminate the influence of pre-existing differences between groups. Because one intact class represented the EG and the other represented the CG, the treatment condition was completely confounded with class membership. Thus, baseline non-significance cannot completely rule out unmeasured class-level differences as alternative explanations for the observed posttest differences. In addition, the instructional program lasted 11 weeks, which may have been insufficient to capture longer-term instructional effects. Moreover, no formal fidelity measure was used to quantify the consistency of peer-tutoring implementation across the 11-week intervention.

Additionally, because identical writing tasks and scoring criteria were used for both the pretest and posttest, potential practice or task-familiarity effects cannot be entirely ruled out. Accordingly, the observed improvements should be interpreted with caution, as they may partly reflect familiarity with the repeated task rather than generalized development in EFL writing competence. Future research should employ parallel or counterbalanced writing tasks to minimize potential task-familiarity effects. In addition, future research involving multiple classes in each condition and randomized assignment of classes to treatment conditions would further strengthen the internal validity of the findings.

Finally, the participants are from a single higher education institution in eastern China, so the generalizability of the findings might be limited. Future studies are recommended to conduct cross-institutional validation to strengthen the external validity of the results.

### Implications

Theoretically, the fact that increased WSE is correlated with increased writing competence is consistent with and extends the use of Bandura’s [4] social cognitive point of view within peer-supported EFL writing situations. From an instructional point of view, EFL writing teachers should include peer-assisted learning strategies and collaborative writing exercises in their teaching plans. These pedagogical approaches create a favorable learning context that helps students build greater confidence in their abilities while improving their writing. In teachers’ pedagogical practice, there should be guidance on how to apply peer-assisted collaborative writing.

Overall, if conducted well, the integration of peer tutoring and collaborative writing practices may provide a supportive context for EFL students to develop their WSE, which might enhance their writing competence.

## Conclusion

The present study provides empirical evidence suggesting that peer tutoring, integrated with collaborative writing activities, is associated with higher levels of Chinese EFL students’ WSE and improved writing competence. Compared with the CG, the EG demonstrated greater gains in both WSE and writing competence. In addition, the increased WSE was positively associated with writing competence. These findings suggest that improvements in writing competence were accompanied by increased WSE and are consistent with theoretical explanations involving peer scaffolding and other psychological processes. However, these processes were not directly measured and should be examined in future research.

## Supporting information

S1 File Inclusivity-in-global-research questionnaire. (DOCX)

S2 File Complete WSE questionnaire. (DOCX)

S3 File Experimental group teaching plan. (DOCX)

S4 File Control group teaching plan. (DOCX)
