# Supplementary material for: A psychological perspective on peer tutoring in EFL collaborative writing in Chinese higher education contexts
Source: PLoS One. 2026 Nov 6;21(11):e0362263. doi: 10.1371/journal.pone.0362263 (PMC13742953; doi:10.1371/journal.pone.0362263)
Supplement: Supplementary file 1 [file pone.0362263.s001.docx]

Inclusivity in global research

PLOS’ policy on inclusivity in global research aims to improve transparency in the reporting of research performed outside of researchers’ own country or community and ensures that PLOS publications reporting global research adhere to high standards for research ethics and authorship. Authors of relevant research articles may be asked to complete the questionnaire below, which outlines ethical, cultural, and scientific considerations specific to inclusivity in global research. This questionnaire may be requested when researchers have travelled to a different country to conduct research, if research uses samples collected in another country, research with Indigenous populations or their lands, or if research is on cultural artefacts. Researchers travelling to another country solely to use laboratory equipment will not normally be required to complete the questionnaire. However, the questionnaire can be requested at the journal’s discretion for any submission – if you have been requested to complete this questionnaire by the PLOS journal you submitted to, please do so.

Please complete the questionnaire below and include this as a Supporting Information file with your manuscript. Note that if your paper is accepted for publication, this checklist will be published with your article in the supporting information files. Please ensure that you reference the checklist in the main body of your manuscript. We suggest adding a subsection ‘Inclusivity in global research’ to your Methods section and adding the following sentence: “Additional information regarding the ethical, cultural, and scientific considerations specific to inclusivity in global research is included in the Supporting Information (SX Checklist)”

The questions have been designed to be applicable to a wide range of study types, and there are subsections for both human subjects research and non-human subjects research. If any of the questions are not relevant to your research please mark them as “N/A” as appropriate.

**Ethical considerations, permits and authorship**

*This section is applicable to all research types.*

Provide details as to who granted permissions and/or consent for the study to take place in the Methods section of your manuscript. This should include the names of **all** ethics boards, governmental organizations, community leaders or other bodies that provided approval for the study. If individuals provided approval refer to these people by their role or title but do not list their name(s).

Reported on page number: Page 8 (Lines 14-17)

If there were any deviations from the study protocol after approval was obtained please provide details of these changes in the Methods section of your manuscript.
Did this study involve local collaborators that are residents of the country where the research was conducted or members of the community studied? If you do not have any authors from said communities, please provide an explanation for this below.

No deviations from the approved study protocol were made after ethical approval.

This study did not include local collaborators as co-authors from the community where the research was conducted. The research was implemented within a higher education institution through formal institutional arrangements. All data collection procedures were conducted in coordination with the course instructors at the participating university to ensure smooth implementation of the study.

Everyone listed as an author should meet PLOS’ criteria for authorship and all individuals who meet these criteria should be included in the author byline, rather than the acknowledgements. For further information please see the journal’s Authorship Policy.

**Human subjects research (e.g. health research, medical research, cross-cultural psychology)**

Did you obtain written informed consent from a representative of the local community or region before the research took place? How did you establish who speaks for the community? Details of written informed consent obtained from study participants should be reported separately in the Methods section of your manuscript.

How did members of the local community provide input on the aims of the research investigation, its methodology, and its anticipated outcome(s)?

This study did not involve formal community participation in the development of the research aims, methodology, or anticipated outcomes. The research design was developed by the research team based on established literature and pedagogical frameworks in EFL writing instruction. However, course instructors at the participating university provided practical input to ensure the feasibility of classroom implementation within the institutional teaching context. All research procedures were finalized under institutional ethical approval.

Written ethical approval was obtained from the Ethics Committee of the Institute of Neuroscience and Cognitive Psychology, Anhui Polytechnic University (AHPU-SLS-2025-003) prior to the study. The institutional ethics committee, operating under formal university governance procedures, served as the authorized body representing the research setting. Therefore, no separate community or regional representative consent was required. Written informed consent was obtained from all individual participants, and details of participant consent are reported in the Methods section of the manuscript.

When engaging with the local community, how did you ensure that the informed consent documents and other materials could be understood by local stakeholders?

All participant-facing materials, including informed consent forms and study instructions, were provided in clear Chinese, which is the participants’ native language. To ensure clarity and accessibility, the documents were written in non-technical language and reviewed by course instructors prior to distribution. Participants were given the opportunity to ask questions before providing written informed consent, ensuring full understanding of the study purpose and procedures.

Yes. The findings of this study will be made available to stakeholders at the participating university in an accessible and understandable format. A non-technical summary of the results will be prepared in Chinese and shared with the School of Foreign Studies and participating instructors. In addition, a brief presentation or report may be provided upon request to support teaching improvement and curriculum development. Published articles arising from the study will also be shared with the institution for academic reference.

Will the findings of the research be made available in an understandable format to stakeholders in the community where the study was conducted (e.g. via a presentation, summary report, copies of publications, etc.)? Please provide details of how this will be achieved.

**Non-human subjects research using specimens/ animals collected as part of the study, or those housed in archival collections. Examples include archaeology, paleontology, botany and zoology.**

Did the permission you obtained from a local authority to perform the study include an agreement on access to outputs and benefit sharing? This may include procedures to enable fair distribution of the benefits and resources arising from the research performed. Please include any details of Prior Informed Consent and Benefit Sharing Agreements obtained. These may be required by field-specific regulations, for example the Convention on Biological Diversity (CBD) and the associated Nagoya Protocol.

Not applicable. This study did not involve non-human subjects or biological resources.

If the material used in your study was imported, please A) provide the year it was imported and B) indicate whether permits were obtained to import/export the materials used, C) provide details of any permits obtained. If this information is not available, please indicate this.

Not applicable, as this study did not involve imported materials.

If you used archival specimens, please state how the material used in your study was acquired by the institute it is held in and provide details of any permits obtained for the original excavations/ sample collection. If this information is not available, please indicate this.

Not applicable, as this study did not involve archival specimens or excavated materials.

How was the potential cultural significance of the materials collected in your study to local communities considered in your research design? Were Indigenous peoples and/or local researchers and institutions involved with archaeological excavations / collection of specimens? If so, please provide a description of their involvement.

Not applicable, as this study did not involve cultural materials, archaeological excavations, or biological specimens, and no Indigenous peoples or local communities were involved in material collection.

If your manuscript includes photographs of human remains please indicate whether authors obtained permission from descendants or affiliated cultural communities to do so.

Not applicable, as this study does not include photographs of human remains.
