# Supplementary material for: A psychological perspective on peer tutoring in EFL collaborative writing in Chinese higher education contexts
Source: PLoS One. 2026 Nov 6;21(11):e0362263. doi: 10.1371/journal.pone.0362263 (PMC13742953; doi:10.1371/journal.pone.0362263)
Supplement: Supplementary file 2 [file pone.0362263.s002.docx]

**Pre- and Post-intervention WSE Questionnaire**

The information you provide will contribute towards a better understanding of how to help you to improve proficiency level in English writing. Your response will be treated in confidence and your cooperation in participating in this study is greatly appreciated.

**Instructions:**

1. Please attempt to answer **ALL** the questions.

2. Please fill in your own response(s) in the spaces provided wherever the listed responses are inappropriate or insufficient.

**PART I: Demographic Information**

1. Age:	……………………………………………..

2. Years of English learning: ………………………

Tick in the appropriate space.

3. Gender:

Male Female

4. State the grades you obtained in English.

| Subject | Scores |
| --- | --- |
| NMET |  |
| CET-4 |  |
| CET-6 |  |

**PART II: Writing Self-Efficacy (WSE)**

Indicate your opinion on English writing performance by ticking the appropriate column

1 = Strongly disagree; 2 = Disagree; 3 = Somewhat disagree; 4 = Neither agree or disagree;

5 = Somewhat agree; 6 = Agree; 7 = Strongly agree

| **Dimensions** | **No.** | **Items** | **1** | **2** | **3** | **4** | **5** | **6** | **7** |
| --- | --- | --- | --- | --- | --- | --- | --- | --- | --- |
| WSE in content | 1 | I can make reasonable choices and support my viewpoint. |  |  |  |  |  |  |  |
|  | 2 | I can express my views and information clearly and explicitly. |  |  |  |  |  |  |  |
|  | 3 | I can use writing resources such as libraries, the internet, etc., to collect information and support my writing content. |  |  |  |  |  |  |  |
|  | 4 | I can evaluate and improve my writing content critically. |  |  |  |  |  |  |  |
|  | 5 | I can continuously improve and refine my writing content during the writing process. |  |  |  |  |  |  |  |
| WSE in organization | 6 | I can follow a logical order to ensure the coherence and rationality of the article’s content. |  |  |  |  |  |  |  |
|  | 7 | I can use appropriate punctuation and spaces to express the relationship between sentences and paragraphs, making the article more straightforward. |  |  |  |  |  |  |  |
|  | 8 | I can use transitional words and sentences to connect paragraphs and sentences, making the article more fluent. |  |  |  |  |  |  |  |
|  | 9 | I can evaluate and modify an article’s logical structure to make it more rigorous and persuasive. |  |  |  |  |  |  |  |
| WSE in language use | 10 | I can understand and apply advanced grammar structures, such as independent nominative and inverted sentences, to enhance the expressiveness and artistry of the article. |  |  |  |  |  |  |  |
|  | 11 | I can understand and use advanced vocabulary and complex sentence structures to enhance the depth and breadth of the article. |  |  |  |  |  |  |  |
|  | 12 | I can identify and correct complex grammar errors, such as connecting clauses and emphasizing the use of sentence structures. |  |  |  |  |  |  |  |
|  | 13 | I can use basic grammar rules correctly and avoid common grammar errors such as subject-verb inconsistency, tense errors, etc. |  |  |  |  |  |  |  |
|  | 14 | I can check and correct vocabulary errors and inappropriate wording to ensure the accuracy and standardization of the article. |  |  |  |  |  |  |  |
|  | 15 | I can identify and use synonyms to avoid repetition and enhance the article’s novelty. |  |  |  |  |  |  |  |
| WSE in mechanics | 16 | I can use appropriate citation methods and formats and comply with academic English writing standards. |  |  |  |  |  |  |  |
|  | 17 | I can follow the norms of English writing, such as spelling, punctuation, and capitalization. |  |  |  |  |  |  |  |
|  | 18 | During the writing process, I can flexibly adjust the format of the article to meet different writing requirements. |  |  |  |  |  |  |  |

**Adapted from:**

Yörüdü, A. E. (2021) An Alternative Multi-Trait Rubric for the Performance-Based Assessment of EFL Writing Proficiency at Bursa Uludag University, School of Foreign Languages

**Note.** The item numbers in this Supporting Information reflect the final 18-item questionnaire and were renumbered for presentation purposes. The original item numbering was retained for the exploratory factor analysis.
