# Supplementary material for: A psychological perspective on peer tutoring in EFL collaborative writing in Chinese higher education contexts
Source: PLoS One. 2026 Nov 6;21(11):e0362263. doi: 10.1371/journal.pone.0362263 (PMC13742953; doi:10.1371/journal.pone.0362263)
Supplement: Supplementary file 3 [file pone.0362263.s003.docx]

**Experimental Group Teaching Plan**

| **Lessons** | **Objectives** | **Activities** | **Approaches** |
| --- | --- | --- | --- |
| Lesson 1  Pre-assessment | 1. Assess students’ initial writing performance and self-efficacy.  2. Train students with peer tutoring skills and group process writing techniques. | 1. Administer the pretest and distribute the pre-survey questionnaire.  2. Group learners based on responses to pretest and pre-survey.  3. Train students with peer tutoring sub-skills: supporting and accepting, paraphrasing and clarifying peers’ contributions,  correcting peers, criticising ideas without criticizing people, expanding ideas, integrating ideas, asking for help, asking for justification, coming to a consensus, and generating further answers.  4. Discuss the features of collaborative writing. | Pretest (Individual Writing Task), Questionnaire; Group  Discussion; Teacher Explanation; |
| Lesson 2  Introduction to Types of Writing and Narration | 1. Let students understand narration.  2. let students be familiar with peer tutoring in writing narration.  3. Cover planning and idea generation in the process writing of “The Mysterious Package”. | 1. Define and explain narration as a type of writing.  2. Guide students through planning and generating ideas for narrative writing of “The Mysterious Package”. | Lecture; Group Discussion; Peer tutoring; Collaborative Writing Task |
| Lesson 3  Writing Narration (1) | 1. Focus on the translating stage of process writing.  2. Facilitate drafting of “The Mysterious Package”. | 1. Discuss the translating stage in process writing.  2. Guide students in drafting “The Mysterious Package”. | Lecture; Group Discussion; Peer Tutoring; Collaborative Writing Task |
| Lesson 4  Writing Narration (2) | 1. Cover editing and revising stages of process writing.  2. Facilitate group revising and finalizing of “The Mysterious Package”. | 1. Explain the editing and revising stages in process writing. Use peer-editing skills concerning content, organization, language use and mechanics.  2. Exchange peer feedback on drafts.  3. Facilitate collaborative revising sessions.  4. Guide students in writing the final draft. | Lecture; Group Discussion; Peer Tutoring; Collaborative Writing Task |
| Lesson 5  Writing Description (1) | 1. Let students understand the description.  2. Plan and generate ideas for “The University Campus” | 1. Discuss the characteristics of descriptive writing.  2. Compare description with narration.  3. Introduce the topic “The University Campus”.  4. Plan and generate ideas for the descriptive essay. | Lecture, Group Activity, Peer Tutoring |
| Lesson 6  Writing Description (2) | Process writing for description (Translating, Editing, and Revising) | 1. Understand the stages of translating, editing, and revising in process writing.  2. Guide students in translating their ideas into written text.  3. Provide support for structuring their descriptive essays.  4. Encourage peer feedback and discussion.  5. Edit and revise drafts based on peer feedback. Use peer-editing skills concerning content, organization, language use and mechanics. | Group Work, Peer Tutoring, Collaborative Writing |
| Lesson 7  Writing  Exposition: Comparison and Contrast (1) | 1. Let students understand comparison and contrast.  2. Plan and generate ideas for “Generation X and Generation Z”. | 1. Discuss the characteristics of comparison and contrast writing.  2. Introduce the topic “Generation X and Generation Z”.  3. Plan and generate ideas for the comparison and contrast essay. | Lecture, Group Activity, Peer Tutoring |
| Lesson 8  Writing Exposition: Comparison and Contrast (2) | Process writing for comparison and contrast (Translating, Editing, and Revising) | 1. Understand the stages of translating, editing, and revising in process writing.  2. Guide students in translating their ideas into written text.  3. Encourage peer feedback and discussion.  4. Edit and revise drafts based on peer feedback. Use peer-editing skills concerning content, organization, language use and mechanics. | Group Work, Peer Tutoring, Collaborative Writing |
| Lesson 9  Writing Argumentation (1) | 1. Let students understand argumentation.  2. Plan and generate ideas for “Should We Set off Fireworks”. | 1. Discuss the characteristics of argumentative writing.  2. Introduce the topic “Should We Set off Fireworks”.  3. Plan and generate ideas for the argumentative essay. | Lecture, Group Activity, Peer Tutoring |
| Lesson 10  Writing Argumentation (2) | Process writing for argumentation (Translating, Editing, and Revising) | 1. Understand the stages of translating, editing, and revising in process writing.  2. Guide students in translating their ideas into written text.  3. Encourage peer feedback and discussion.  4. Edit and revise drafts based on peer feedback. Use peer-editing skills concerning content, organization, language use and mechanics. | Group Work, Peer Tutoring, Collaborative Writing |
| Lesson 11  Post-assessment | 1. Evaluate learning outcomes.  2. Gather feedback on course effectiveness.  3. Assess individual writing skills and comprehension. | 1. Administer post-test: Introduce a book and explain why it is worth reading.  2. Conduct a post-intervention questionnaire. | Post-test (Individual Writing Task), Questionnaire, |
