# Supplementary material for: A psychological perspective on peer tutoring in EFL collaborative writing in Chinese higher education contexts
Source: PLoS One. 2026 Nov 6;21(11):e0362263. doi: 10.1371/journal.pone.0362263 (PMC13742953; doi:10.1371/journal.pone.0362263)
Supplement: Supplementary file 4 [file pone.0362263.s004.docx]

**Control Group Teaching Plan**

| **Lessons** | **Objectives** | **Activities** | **Approaches** |
| --- | --- | --- | --- |
| Lesson 1  Pre-assessment | 1. Assess students’ initial writing performance and self-efficacy.  2.Let students understand the requirements of this course. | 1. Administer pretest and distribute the pre-survey questionnaire.   2. Introduce course content and objectives.  3. Explain the structure of the course and expectations. | Test, Questionnaire, Lecture |
| Lesson 2  Introduction to Types of Writing and Narration | 1. Let students understand narrative writing. 2. Let students know the differences between narration and other types of writing. | 1. Explain the characteristics of narration.  2. Analyze examples of narrative writing.  3. Discuss the differences between narration and other types of writing. | Lecture, Teacher-Led Analysis |
| Lesson 3  Writing Narration (1) | Let students be familiar with relevant grammar, vocabulary, and sentence structures for narration. | 1. Teach relevant grammar rules and vocabulary for narrative writing.  2. Provide examples of effective sentence structures in narratives.  3. Have students practice writing sentences and short paragraphs using the taught structures. | Lecture, Individual Work |
| Lesson 4  Writing Narration (2) | Let students acquire skills in writing and improving narration (“The Mysterious Package”) | 1. Focus on narrative writing techniques (e.g., plot development, characterization).  2. Have students draft their own narrative based on the topic “The Mysterious Package”.  3. Provide teacher feedback on students’ drafts, focusing on grammar, vocabulary, and sentence structures.  4. Guide students in revising their drafts based on the feedback. | Lecture, Individual Work |
| Lesson 5  Writing Description (1) | Let students understand descriptive writing | 1. Explain the characteristics of descriptive writing.  2. Analyze examples of descriptive passages.  3. Discuss the differences between description and other types of writing. | Lecture, Teacher-Led Analysis |
| Lesson 6  Writing Description (2) | Let students freely use relevant grammar, vocabulary, and sentence structures for description | 1. Teach relevant grammar rules and vocabulary for descriptive writing.  2. Provide examples of effective sentence structures in descriptions.  3. Have students practice writing sentences and short paragraphs using the taught structures.  4. Provide teacher feedback on students’ practice writing, focusing on grammar, vocabulary, and sentence structures. | Lecture, Individual Work |
| Lesson 7  Writing Exposition: Comparison and Contrast (1) | Let students understand comparison and contrast in writing exposition | 1. Explain the structure and characteristics of comparison and contrast essays.  2. Analyze examples of comparison and contrast writing (“Generation X and Generation Z”).  3. Discuss the differences between comparison and contrast and other types of writing. | Lecture, Teacher-Led Analysis |
| Lesson 8  Writing Exposition: Comparison and Contrast (2) | Let students freely use relevant grammar, vocabulary, and sentence structures for comparison and contrast | 1. Teach relevant grammar rules and vocabulary for comparison and contrast essays.  2. Provide examples of effective sentence structures in such essays.  3. Have students practice writing sentences and short paragraphs using the taught structures.  4. Provide feedback on students’ practice writing, focusing on grammar, vocabulary, and sentence structures. | Lecture, Individual Work |
| Lesson 9  Writing Argumentation (1) | Let students understand argumentation argumentative writing. | 1. Explain the structure and characteristics of argumentative essays.  2. Analyze examples of argumentative writing (“Should We Set off Fireworks”).  3. Discuss the differences between argumentation and other types of writing. | Lecture, Teacher-Led Analysis |
| Lesson 10  Writing Argumentation (2) | Let students freely use relevant grammar, vocabulary, and sentence structures for argumentation. | 1. Teach relevant grammar rules and vocabulary for argumentative essays.  2. Provide examples of effective sentence structures in such essays.  3. Have students practice. | Lecture, Individual Work |
| Lesson 11  Post-assessment | 1. Evaluate learning outcomes.  2. Assess individual writing skills. | 1. Administer post-test: Introduce a book and explain why it is worth reading.  2. Conduct a post-intervention questionnaire. | Post-test (Individual Writing Task), Questionnaire, |
